# Morbidly Obese Woman Unaware of Pregnancy until Full-Term and Complicated by Intraamniotic Sepsis with Pseudomonas

**DOI:** 10.1155/2007/51689

**Published:** 2008-02-06

**Authors:** H. Muppala, J. Rafi, I. Arthur

**Affiliations:** ^1^Department of Obstetrics and Gynaecology, Royal Albert Edward Infirmary, Wigan Lane, Wigan WN1 2NN, Lancashire, UK; ^2^Department of Obstetrics and Gynaecology, Delaunays Road, Crumpsall M8 5RB, Manchester, UK; ^3^Women's Health Directorate, Blackpool Victoria Hospital, Whinney Heys Road, Blackpool, Lancashire FY3 8NR, Lancashire, UK

## Abstract

A 32-year-old Caucasian woman of body mass index (BMI) 46 presented with urinary symptoms to accident and emergency (A&E). Acute pyelonephritis was the diagnosis. Transabdominal scan revealed a live term fetus. Both the partners were unaware of the ongoing pregnancy until diagnosed. She underwent emergency cesarean under general anaesthesia (GA) for nonreassuring CTG, severe chorioamnionitis, and moderate preecclampsia. A live male baby weighing 4400 grams delivered in poor condition. Placental tissue on culture exhibited scanty growth of *pseudomonas aeruginosa*. Chorioamnionitis due to *pseudomonas* is rare, with high neonatal morbidity and mortality. It is mostly reported among preterm prelabor rupture of membranes (PPROM). Educating the community especially morbidly obese women if they put on excessive weight or with irregular periods should seek doctor's advice and exclude pregnancy. For the primary care provider, it is of great importance to exclude pregnancy in any reproductive woman presenting with abdominal complaints. This case also brings to clinicians notice that *pseudomonas* can be community-acquired and can affect term pregnancies with intact or prolonged rupture of membranes.

## 1. CASE REPORT

A 32-year-old Caucasian woman with BMI 46 presented to “A&E” with dysuria, urinary frequency, and incontinence, along with bilateral loin tenderness and vomiting. Her initial observations were as follows: pulse 135, BP 151/114, and temp. 36.6. Urine dipstick revealed 3+ protein, blood, leucocytes, and ketones. Liver and renal function tests, uric acid, amylase, random blood sugar, and clotting screen were within normal range; Hb 10.0, white cell count of 26.2 with Neutrophils at 23.32, platelets 454, and C*ß*reactive protein >180 mg/L. Haematology report also showed microcytic hypochromic blood picture. Midstream urine was sent for culture and sensitivity. The immediate impression was that of acute pyelonephritis and treatment commenced with IV Cefuroxime and surgical review requested. On abdominal examination, a large, mobile, nontender mass was palpated with positive urine pregnancy test.

Subsequently, transabdominal scan revealed a single live term fetus with cephalic presentation, reduced liquor (amniotic fluid index of 5), effective foetal weight of 3.9 kg, and with right lateral placenta. She was then transferred to delivery suite. This was her first pregnancy and both partners were unaware of the ongoing pregnancy until diagnosed. She gave vague history of constant vaginal discharge. Until then, she was on continuous contraceptive pills. Past medical and surgical history suggested nothing significant, and there was no ongoing psychiatric illness. Interestingly, she presented to general practitioner (GP) at least twice with complaints of backache and urinary symptoms prior to delivery. Although GP recommended treatment for UTI with trimethoprim, she did not take them due to those medications causing sickness.

Upon admission to delivery suite, her observations were pulse 127, BP 160/102, and temp. 37.6 with 3 in 10 uterine contractions. Also noted were 2+ generalised oedema, 3+ proteinuria, and normal reflexes. Cardiotocograph reading showed baseline tachycardia at 170, variability <5, no accelerations, and variable decelerations. Vaginal examination revealed cervix 5 cm dilated, 1+ caput, vertex −2 station, and malodorous vaginal discharge on rupture of membranes. She underwent emergency caesarean section under GA for fetal distress, severe chorioamnionitis, and moderate preecclampsia. There was delay in starting the caesarean due to difficulty in securing the IV access, three failed attempts at spinal, failed catheterisation as pus pouring from the vagina, and due to body habitus.

A live male baby weighing 4400 grams was delivered and handed over to paediatrician. Intraoperative findings revealed offensive liquor in the form of frank pus draining from the uterus, J-shaped uterine incision due to difficulty in delivering the shoulders, pus draining from foetal nose and mouth, normal looking bilateral tubes and ovaries, and a corrugated catheter drain left in the peritoneal cavity due to peritoneal contamination of uterine contents. Vagina was swabbed and Foleys catheter was inserted into bladder. Estimated blood loss was at 700 mL.

Placental and cervical swabs were taken for culture and sensitivity, and placenta was sent for histology. Postoperatively IV Augmentin continued for 48 hours and then oral for 21 days, Labetalol 200 mg orally twice daily for 2 weeks, iron supplements for next 3 months, subcutaneous Tinzaparin 4500 IU for 5 days, and leg stockings until she mobilised. She also received 3 units of blood transfusion in view of her Hb 6.1 gm/dL. All her observations including haematological and biochemical parameters improved over 72 hours. Drain removed on Day six. She was rubella immune and negative for Hepatitis B, Syphilis, and HIV 1 and 2. No pathogenic organisms isolated from urine specimen, placental, and cervical swabs. Placental tissue on culture exhibited scanty growth of *Pseudomonas aeruginosa* sensitive to Ceftazidime, Ciprofloxacin, Gentamicin, Imipenem, and Tazocin. She recuperated uneventfully and discharged home on Day 16.

The APGAR scores for the baby at 1, 5, 10, and 20 minutes were 2, 3, 5, and 7, respectively. Baby was in poor condition at birth and had to be resuscitated and admitted to Special Care Baby Unit. Arterial and venous blood gas pH values were 7.217 and 7.130, respectively. Similarly arterial and venous base deficits were −8.9 and −7.9 mmol/L. Baby developed septicaemia due to *Staphylococcus epidermis* and were treated with antibiotics for seven days. There were developmental delays and a diagnosis of Grade 3 hypoxic ischaemic encephalopathy was made. Baby was discharged home on Day 22 after establishing bottle feeds.

## 2. DISCUSSION

*Pseudomonas aeruginosa* is primarily a nocosomial pathogen, Gram-negative, aerobic, rod-shaped bacterium [1]. Among several commensal flora in the vagina, *Pseudomonas aeruginosa* accounts for nearly 2% [2]. Chorioamnionitis due to *Pseudomonas aeruginosa* is rare and its importance is due to its virulence, with high neonatal morbidity and mortality [3–5]. *Pseudomonas chorioamnionitis* are mostly reported among women with PPROM and in their babies [6, 7]. One of the risk factors identified in literature is prolonged antibiotic therapy in these women [8]. In the case presented, reported infection might have been either community-acquired or opportunistic, due to altered local vaginal flora or generalised immune suppression with haematogenous dissemination. Chorioamnionitis per se could be due to prolonged rupture of membranes or consequent upon urinary tract infection although infection with intact membranes cannot be ruled out due to its high virulence. She gave no history suggestive of prolonged antibiotic therapy. Low socioeconomic status, poor maternal nutrition, and the fact no antenatal care may all have predisposed to this highly virulent pathogenic chorioamnionitis. Gestational diabetes is not ruled out. Consequently, poor outcome of the baby at delivery is the result of severe chorioamnionitis leading to unsafe intrauterine milieu. The *Staphylococcus epidermis* sepsis in the neonate could have been acquired following delivery, especially with interventions such as mechanical ventilation and intubation. Our patient was at risk of septicaemia, shock, disseminated intravascular coagulopathy, adult respiratory distress syndrome, and multiorgan failure [9]. Despite the low incidence of maternal morbidity, these women should be regarded as high-risk labor due to increased perinatal morbidity [10]. Negative placental swabs may be due to IV Cefuroxime commenced earlier in A&E for UTI to which the organism was susceptible as revealed in the placental tissue culture. The reason for starting and continuing Augmentin was due to departmental protocol and for its broad-spectrum activity, as well as improvement in patients’ clinical condition.

This case by definition is a true denial of pregnancy. In other words, unconscious denial of pregnancy whereby pregnancy is denied at all levels—physiological, psychological, and social [11]. The prevalence of this kind of pregnancy is 1 out of every 2455 births, wherein the fetus was born without any preceding subjective awareness of pregnancy on the part of the woman [12].

During postnatal period, appropriate counseling had been given with regard to parenting skills and referral made to social services. A named midwife and health visitor had been appointed to provide any assistance that this woman and her baby may require. Recent evidence suggests that psychiatric consultation was rare for women who had denied or concealed their pregnancies, and yet they would often subsequently take responsibility for their infants [13].

It is important to provide multidisciplinary consultant-led care when the patient is in the hospital and liaise with other professionals, such as social services, community midwife, and health care visitors to provide high quality of care to these patients.

It is difficult to understand what led to total ignorance or insensible to pregnancy changes until full term and delivery. Probably social, psychological, physical factors, or all of them had a role to play. Educating the community especially morbidly obese women if they put on excessive weight or with irregular periods should seek doctor's advice and exclude pregnancy. For the primary care provider it is of great importance to exclude pregnancy in any reproductive woman presenting with abdominal complaints.

This case also brings to clinicians notice that Pseudomonas may have been acquired in the community and affect term pregnancies with intact or prolonged rupture of membranes.
